# Targeting a Cross-Reactive Gly m 5 Soy Peptide as Responsible for Hypersensitivity Reactions in a Milk Allergy Mouse Model

**DOI:** 10.1371/journal.pone.0082341

**Published:** 2014-01-09

**Authors:** Renata Curciarello, Paola L. Smaldini, Angela M. Candreva, Virginia González, Gustavo Parisi, Ana Cauerhff, Ivana Barrios, Luis Bruno Blanch, Carlos A. Fossati, Silvana Petruccelli, Guillermo H. Docena

**Affiliations:** 1 Laboratorio de Investigaciones del Sistema Inmune-LISIN, Departamento de Ciencias Biológicas, Facultad de Ciencias Exactas, Universidad Nacional de La Plata, La Plata, Buenos Aires, Argentina; 2 Centro de Estudios e Investigaciones, Departamento de Ciencias y Tecnología Universidad Nacional de Quilmes, Quilmes, Buenos Aires, Argentina; 3 Centro de Investigación y Desarrollo en Fermentaciones Industriales, CINDEFI (UNLP y CONICET) and Instituto de Investigaciones Bioquímicas de Buenos Aires (IIBBA-CONICET), Fundación Instituto Leloir, Buenos Aires, Argentina; 4 Cátedra de Química Medicinal, Departamento de Ciencias Biológicas, Facultad de Ciencias Exactas, Universidad Nacional de La Plata, La Plata, Buenos Aires, Argentina; 5 Centro de Investigaciones y Desarrollo en Criotecnología de Alimentos-CIDCA, Facultad de Ciencias Exactas, Universidad Nacional de La Plata, La Plata, Buenos Aires, Argentina; National Council of Sciences (CONICET), Argentina

## Abstract

**Background:**

Cross-reactivity between soybean allergens and bovine caseins has been previously reported. In this study we aimed to map epitopes of the major soybean allergen Gly m 5 that are co-recognized by casein specific antibodies, and to identify a peptide responsible for the cross-reactivity.

**Methods:**

Cow's milk protein (CMP)-specific antibodies were used in different immunoassays (immunoblotting, ELISA, ELISA inhibition test) to evaluate the *in vitro* recognition of soybean proteins (SP). Recombinant Gly m 5 (α), a truncated fragment containing the C-terminal domain (α-T) and peptides of α-T were obtained and epitope mapping was performed with an overlapping peptide assay. Bioinformatics tools were used for epitope prediction by sequence alignment, and for modelling the cross-recognized soy proteins and peptides. The binding of SP to a monoclonal antibody was studied by surface Plasmon resonance (SPR). Finally, the *in vivo* cross-recognition of SP was assessed in a mouse model of milk allergy.

**Results:**

Both α and α-T reacted with the different CMP-specific antibodies. α-T contains IgG and IgE epitopes in several peptides, particularly in the peptide named PA. Besides, we found similar values of association and dissociation constants between the α-casein specific mAb and the different milk and soy components. The food allergy mouse model showed that SP and PA contain the cross-reactive B and T epitopes, which triggered hypersensitivity reactions and a Th2-mediated response on CMP-sensitized mice.

**Conclusions:**

Gly m 5 is a cross-reactive soy allergen and the α-T portion of the molecule contains IgG and IgE immunodominant epitopes, confined to PA, a region with enough conformation to be bound by antibodies. These findings contribute to explain the intolerance to SP observed in IgE-mediated CMA patients, primarily not sensitised to SP, as well as it sets the basis to propose a mucosal immunotherapy for milk allergy using this soy peptide.

## Introduction

Food allergy is an increasing health care concern. Cow's milk protein (CMP) is the leading cause of food allergy in children younger than 3 years in many regions, including our country [1], [2]. This is reflected by most of the position papers and guidance with statements and recommendations for food allergy that are focused on CMP allergy (CMA) [3].

If symptoms were relevant and CMA were suspected, a diagnostic dietary elimination of CMP should be initiated for a certain period of time (days to 4 weeks) [4], [5]. Once a diagnosis of allergy to milk proteins has been confirmed, it is important to avoid CMP and to ensure a healthy balanced diet that promotes an adequate child growth and development. Depending on the age and other food allergies a restriction or milk free diet is indicated as therapy.

Soy-based formulas have been available for almost 100 years and have been widely used as a milk substitute in children intolerant to cow's milk protein-based formula [6]. However, there are some indications and contraindications of soy formulas since gastrointestinal reactions, including hypersensitivity, have been described for more than 50 years [7], and because of the nutritional quality of soy proteins [8]. More recently, it has been reported that a small proportion of children with IgE and non IgE-mediated milk allergy develop a clinical intolerance to soy-protein-based formula during the restriction treatment, which may be explained by cross-reactivity once milk contamination has been discarded [6], [9], [10]. We focused our work in investigating the cross-recognition between CMP and soy components. We identified bovine caseins, the main allergens of milk [11], [12], and the soybean Gly m 6 G4 as cross-reactive allergens [13]–[15]. The analysis of a soy variety that naturally lacks the Gly G4 led us to detect that a residual cross-reactivity may lie on Gly m 5.0101, the α subunit of the β-conglycinin. Besides, both seed proteins have been characterized as allergens in patients with IgE-mediated atopic dermatitis [16], food allergy [17], [18], asthma [19] and anaphylaxis [20], [21]. Therefore, we propose that the clinical intolerance observed in a restricted proportion of milk allergic patients, with documented IgE specific to milk proteins, may be due to the presence of these relevant cross-reactive allergens in soy protein-based formulas.

In this study, we immunochemically and biologically characterized the α subunit of β-conglycinin, and a C-terminal fragment called α-T, containing putative cross-reactive epitopes with bovine caseins. Using bioinformatic, immunochemical and biological tools we demonstrated the presence of B epitopes on this molecule, and we identified a polypeptide that contains critical epitopes for cross-recognition. The results presented here may shed some light to understand the concomitant soy allergy that occurs in infants with IgE-mediated CMA, and not primarily sensitised to soy proteins. However, and more challenging for us, the identification of cross-reactive B and T epitopes would set the basis to propose a soy-based immunotherapy for milk and soy allergies, taking advantage of the lower immunogenicity of soy proteins.

## Methods

### Protein Extracts

Cow's milk protein extract was prepared from skimmed dry milk (Svelty, Nestle). Proteins were extracted with phosphate saline buffer pH 7.4 (10 mg/ml) and filtered. Soy protein (SP) extract was prepared from *Glycine max*. L merr genotype Asgrow seeds as described previously [14]. Briefly, proteins were extracted from crushed seeds with 0.01N NaHCO_3_ at 90°C. The extract was centrifuged (2,500×g-20 min at room temperature) and lipids were extracted with chloroform (overnight at 4°C). Finally, the extract was dialyzed against distilled water. The total protein content was quantified with the Bicinchoninic acid kit (Pierce BCA Protein Assay), using bovine serum albumin (BSA) as standard.

### Production of the α Subunit of β-conglycinin and α-T Fragment in *Escherichia coli*

The cDNA coding sequence for α (GenBank: X17698) was obtained by PCR amplification of cDNA library from developing *Glycine max* L. Merr. seeds [22]. pET-mα and pET α-T [22] were transformed into *Escherichia coli* strain BL21(DE3) for protein expression of α subunit of β-conglycinin (α) and α-T, its truncated polypeptide containing three alpha helix and one cupin domain typical of the cupin superfamily [23]. Production and purification of the recombinant proteins are described elsewhere [14].

### PA and PN Peptide Constructs, Expression and Purification

The oligonucleotide primers used for expression of peptides A and N (PA and PN, amino acids 357–418 and amino acids 438–499 of the pre-pro α subunit, respectively) were: F-pepA (CACCCTGTTTAGTAGAGAGGAAG) and R-pepA (TCACTTGTTGGAGTAGATGGG), F-pepN (CACCATCTTCCTCAGTATTGTGG) and R-pepN (TCATTCGGCTCTATATTTCCGC). Amplified PCR products were cloned directionally into pENTR/D TOPO (Life Technology, S.A. Buenos Aires, Argentina), and then transferred to pDEST destination vector for expression [24]. E. coli BL21 Codon Plus competent cells transformed with the construct “pDEST His-PA” and “pDEST His-PN”, were induced for protein expression. Recombinant proteins were purified as described previously [14] and protein concentration was determined by BCA protein assay.

### Production of Polyclonal and Monoclonal Antibodies

CMP- and SP-specific polyclonal antisera obtained in rabbit using complete or incomplete Freund's Adjuvant, and a specific α-casein monoclonal antibody (1D5 mAb) were all obtained as described in Docena et al [11]. 1D5 mAb was biotinylated for the SPR biosensor assay. Briefly, 1D5 mAb (1 mg/ml in 0.1M borate buffer, pH 8.8) was incubated with 10 mg/ml solution of N-hidroxisuccinimide-biotin in DMSO (250 µg∶1 mg of biotin∶mAb ratio) for 4 h at room temperature. The biotinylated mAb was desalted by dialysis against PBS and biotinylation was assessed by ELISA using alkaline phosphatase-conjugated streptavidin (Sigma Aldrich, St Louis, MO). For this assay wells were coated with CMP (1 µg/well), and after blocking, dilutions (1∶100 to 1∶100,000) of the biotinylated-1D5 mAb were added, followed by the alkaline phosphatase-conjugated streptavidin (1∶4,000). Color was developed with *p*-nitrophenylphosphate in buffer pH 9.6, and optical density (OD) at 405 nm was measured in an ELISA reader (Sirio S SAECS, Roma, Italy).

### Patient Sera Used for *In Vitro* Characterization of the Allergen Content

Sera obtained from 15 atopic children (ages ranging from 3 months to 10 years, mean age: 4.5 years) with documented IgE-mediated CMA by history, and recruited for other studies, were used. Double blind placebo-controlled food challenge was not done since it is not performed in Argentina for diagnosis. Patients showed clinical pictures of immediate reactions associated to the ingestion of milk or milk-derived products (vomiting, diarrhoea, dermatitis, bronchospasm, urticaria and rhinorrhea). Histories showed no evidence of soybean sensitization. CMP-specific IgE antibodies were detected by immunoblotting, ELISA and EAST (Class 2 or higher) as described previously [12]. Skin prick test with commercial extracts (CMP and common environmental allergens) (Alergo Pharma, Buenos Aires, Argentina) was performed. Histamine (10 µg/ml) and PBS (pH 7.4) were used as positive and negative control, respectively.

Besides, sera from 20 subjects (age- and sex-matched) with no history of food allergy, no clinical manifestations after ingestion of CM, with normal level of serum total IgE according to age, and no specific serum IgE antibodies were used as negative controls.

### SDS PAGE and Immunoblotting

SDS-PAGE and immunoblotting were performed as described before [14]. Samples prepared in sample buffer were loaded on gels (10–20 µg/lane for CMP or SP, or 7–10 µg/lane for the recombinant proteins) and electrotransferred onto nitrocellulose membranes. Membranes were probed with the 1D5 mAb (1∶1,500 in TBS-Tween 20), cow's milk-specific rabbit polyclonal antiserum (1∶1,000 in TBS-Tween 20) for 1 h at 37°C, or with patient sera (1∶4 in blocking buffer) overnight at 4°C, as primary antibody, followed by the appropriate conjugated secondary antibody which were: biotinylated goat anti-mouse immunoglobulin G (1∶3,000, Chemicon Int., Temecula, CA), biotinylated mouse monoclonal antibodies specific to rabbit immunoglobulins (1∶3,000, Sigma-Aldrich, St Louis, MO) and horseradish peroxidase-conjugated avidin (1∶4,000, Sigma-Aldrich, St Louis, MO), or horseradish peroxidase-conjugated anti-human IgE antibodies (dilution 1∶1,000, Sigma Aldrich, St Louis, MO) for 4 h at 37°C. Protein bands were visualized by enhanced chemiluminescence (ECL Plus, GE Healthcare, Bucks, UK) according to manufacturer's instructions. X-ray-sensitive films (Amersham Hypefilm™ MP, GE Healthcare Limited, Pollarsd Wood, UK) were used and images were acquired with a Hewlett Packard scanner.

### Indirect ELISA for IgE Reactivity

Polystyrene microtiter plates (NUNC, Maxisorp, Denmark) were coated with 1.25 µg/well of CMP, 0.05 µg/well of SP or with 0.05 µg/well of α or α-T, in 50 mM carbonate buffer (pH 9.6). Plates were blocked with 5% horse serum in PBS (pH 7.4), and the ELISA was carried out using the patient or negative control sera as primary antibody, as previously described [11], [14]. Cut-off values were statistically obtained (SEM+2 SD) from the readings of the negative control sera used.

### Sequential Competitive ELISA

ELISA plates were coated overnight with 1 µg/well of CMP and blocked with 5% horse serum in PBS (pH 7.4) for 1 hr at 37°C. CMP-specific rabbit antiserum diluted 1∶200,000 in 3% horse serum in PBS (pH 7.4), or α-casein specific mAb diluted 1∶300,000 in the same buffer, were mixed (1∶1) with different concentrations of inhibitors: CMP, α α-T or bovine serum albumin (BSA) (Sigma Aldrich, St Louis, MO) for 2 h at 37°C. Then 100 µl of this pre-mixed dilution was added in triplicates to the antigen-coated wells and incubated for 30 min at 37°C. Biotinylated mouse monoclonal antibodies specific to rabbit immunoglobulins (1∶3,000, Sigma-Aldrich, St Louis, MO) or biotinylated goat anti-mouse immunoglobulin G (1∶3,000, Chemicon Int., Temecula, CA) were added for 1 h at 37°C. Finally, horseradish peroxidase-conjugated avidin (1∶3,000, Sigma-Aldrich, MO, USA) was added (30 min at 37°C) and color was developed with *o*-phenylenediamine and 30% H_2_O_2_ in 0.1M citrate-phosphate buffer pH 5.0. Optical density was measured at 492 nm in an ELISA reader. Additionally, 96-well plates were coated with α or α-T (0.1 µg/well) and blocked. CMP-specific polyclonal antiserum diluted 1∶6,000 was used in the inhibition mix with same inhibitors. Then, 100 µl of the pre-mixed dilution was added to the coated wells and developed as described above.

### Cross-reactive Epitope Mapping on α-T

Overlapping 15-mer peptides, frame shifted by five residues, covering the entire aminoacid sequence of α-T were synthesized on a cellulose membrane (PepSpot JPT Peptide Technologies GMBH, Germany). Spots membrane-based immunoassay was carried out by incubation of the blocked membranes with different primary antibodies (pools of sera containing CMP specific IgE, CMP-specific rabbit polyclonal antiserum or 1D5 mAb), followed by the appropriate secondary antibody. Membranes were developed by chemiluminescence as described above.

### Binding Kinetics Analysis Using the BIACORE Assay

Affinity sensor analysis experiments were conducted on an IAsys Plus apparatus (Affinity Sensors, Saxon Hill, Cambridge, U.K.). Streptavidin (Sigma Aldrich, USA) (100 µg/ml in acetate buffer, pH 5.0) was covalently coupled to carboxymethyl dextran sensor chips (Affinity Sensors, Saxon Hill, Cambridge, U.K.) (5 ng of protein per cuvette). Then, a solution of 20 µg/ml in saline buffer of the biotinylated 1D5 mAb (the ligand) was added to the cuvette during 10 minutes, the antibody excess was removed by adding 1M NaCl and 10 mM acetate buffer at pH 4.5. Binding reactions with the ligate were carried out in PBS, 0.05% Tween 20 at 25°C, with constant stirring set up at 90%. Data were collected at intervals of 0.3 sec. Ligate binding to the immobilized ligand was monitored at multiple ligate concentrations, ranging 10-fold below to at least 10-fold above preliminary estimates of equilibrium dissociation constants (K_D_) for each reaction. Kinetic and Scatchard analysis were performed by using the FAST FIT software (Affinity Sensors, Saxon Hill, Cambridge, U.K.).

### Bioinformatics Analysis

Caseins and subunits of soybean β-conglycinin were compared using bioinformatics tools. The sequences of α (gi:9967357: 543 aa), α′ (gi:15425631: 621 aa) and β (gi: 9967359: 416 aa) subunits of β-conglycinin, and αs1- (gi:162792: 214 aa), αs2- (gi:27806963:222 aa), β- (gi:162931:224 aa) and κ- (gi:1228078: 190 aa) caseins were retrieved from the GenBank database. Several epitope prediction methods available in the B Cell Epitope Prediction Tools at the Immune Epitope Database (IEDB) [25] (http://www.immuneepitope.org) were applied. These methods included Chou & Fasman Beta-Turn Prediction [26], Emini Surface Accessibility Prediction [27], Karplus & Schulz Flexibility Prediction [28], Kolaskar & Tongaonkar Antigenicity [29], and Parker Hydrophilicity Prediction [30]. Peptides identified as putative epitopes based on these predictions were collected in a database. A second database was built using overlapping peptides (15 residues long) of the cow milk and soybean proteins mentioned above. Both databases were aligned with the BLASTP program using parameters fixed to short peptides (Expect Value = 20,000, Score Matrix = PAM30, Word Size = 2, SEG Filter = off) [31] and a shorter region of α located on the α-T segment, containing most amino acid coincidences with bovine caseins epitopes, and named “Peptide A (PA)”, was selected. We have used MODELLER [32] to build structural models of α protein based on templates found with FFAS03 fold assignment method [33]. Three-D models were assessed using ProsaII [34] and the PyMOL (http://www.pymol.org) was employed to map α-T and PA on the best 3D models obtained. Calculation of the electrostatic potential surface was made with the web-based graphical user interface PBEQ-Solver [35] and the electrostatic map was visualized in PyMOL.

The hit distribution graph was done to represent those similar amino acid positions shared by soybean α, α-T, and bovine casein peptides. The amino acid sequence of 73 peptides of αS1-, αs2-, β- and κ-casein, described as T and/or B epitopes (IEDB, (http://www.immuneepitope.org/), were aligned with α-T sequence by the ClustalW2 server (http://www.ebi.ac.uk/Tools/msa/clustalw2/). The hits represent the accumulation of consensus amino acid in every position of the sequence analyzed. A score of “3” was assigned for conserved aminoacid (identity), “2” for similar, but not identical amino acids (punctuation over 0.5 on Gonnet or PAM 250 matrixes), and “1” for slightly similar amino acids (punctuation below 0.5 on Gonnet or PAM 250 matrixes).

### Secondary Structure Prediction

Bioinformatics studies described above allowed us to find out that the PA fragment has a primary structure alignment with a fragment of bovine α-casein. Different secondary structure prediction servers and softwares were used to analyze both fragments. Prediction programs belong to the Network Protein Sequence Analysis server [36], which contains SOPM, SOPMA, Hierarchical Neural Network, GOR I, GOR III, GOR IV, DPM, DSC, PHD, PREDATOR, SIMPA 96 and Secondary consensus prediction. Other programs such as CFSSP [37], Jpred [38], JUFO 3D [39], NetSurfP and Multivariate Linear Regression Combination were also used. The final secondary structure prediction was the average of all structures obtained with those prediction programs. Besides, the alignments were performed using the BLAST program.

### Mice and Experimental Model of IgE-mediated Food Allergy

#### Sensitization and Antigenic Challenge

Male 6- to 8-week old Balb/c mice were purchased from the School of Animal Sciences, University of La Plata, and kept under pathogen-free conditions with water and commercial diet provided *ad libitum*. Cow's milk sensitization protocol was carried out as previously described [15]. Briefly, mice received 6 weekly intragastric (ig) doses of CMP (20 mg/dose) plus cholera toxin (10 µg/dose) (Sigma Aldrich, St Louis, MO, USA) in a final volume of 200 µl of bicarbonate buffer (sensitization group), or CMP (20 mg/dose) without cholera toxin (control group). Mice were fasted for 2 h before sensitization, and 3% sodium bicarbonate solution was given 30 min before the immunization. Ten days following the last boost mice were ig challenged with different proteins: 20 mg of CMP, 5 mg of SP or 10 mg of OVA (Sigma-Aldrich, St Louis, MO, USA) as an unrelated antigen. Additionally, some animals were sublingual (sl) challenged with 10 µg of purified proteins [β-lactoglobulin (β-lg), α, α-T, PA or OVA]. Symptoms immediately triggered after the oral challenge were scored in a blinded manner by two independent observers.

### In vivo evaluation of the allergic state

#### Assessment of Clinical Signs

Symptoms were observed 30–60 min following the oral challenge, in a blinded fashion by 2 independent researchers, and scored on a scale from 0 to 5. Some animals received sl 10 µg of recombinant soybean proteins, β-lg (positive control), or OVA (unrelated negative control), and elicited symptoms were scored.

#### Cutaneous Tests

Mice were shaved on both flanks and subcutaneously injected with 200 µg of CMP, 400 µg of SP, 10 µg of α, α-T or PA in 50 µl of sterile saline in one flank, and 10 µg of saline in the other flank, as a negative control. Mice were also intravenous injected in the tail vein with 100 µl of 0.1% Evans blue dye (Anedra, Buenos Aires, Argentina). The presence of blue color in the skin 15 to 30 min following the injection was considered a positive cutaneous test.

### In vitro evaluation of the allergic state

#### Specific IgE Detection

Serum specific IgE antibodies were measured by EAST using the method described by Ceska et al [40]. Briefly, cyanogen bromide -activated cellulose paper discs were coupled with CMP (1.75 mg/ml), SP (0.5 mg/ml), or the recombinant proteins (0.2 mg/ml). Discs were blocked with 0.5 mM ethanolamine, and incubated overnight at 4°C with 50 µl of undiluted serum samples. Then, the assay was developed as described by Smaldini et al [15].

#### Specific IgG1 and IgG2a Detection

serum CMP- or SP-specific IgG1 and IgG2a were measured by ELISA as described by Smaldini et al [15]. Briefly, microtitre plates were coated with 1 µg/100 µl of either CMP, SP, α, α-T or PA in carbonate-bicarbonate buffer, pH 9.6. The assay was developed as indicated [15].

#### Cytokine Response of Antigen-stimulated Splenocytes

24 h following the oral challenge mice were killed, spleens were removed and cells were isolated. Splenocytes (4×10^6^ cells/well) were plated in complete medium (RPMI-1640 supplemented with 10% FBS, 100 U/ml penicillin and 100 mg/ml streptomycin), and stimulated for 72 h with CMP (350 µg/ml), SP (200 µg/ml), ConA (5 µg/ml), α (15 µg/100 µl), α-T (15 µg/100 µl) or PA (15 µg/100 µl). Supernatants were collected, and cytokines were quantified by ELISA: IL-5, IFN-γ (Invitrogen, Invitrogen Corporation, USA).

### Ethics Statement

All experimental protocols of this study were conducted in strict agreement with international ethical standards for animal experimentation (Helsinki Declaration and its amendments, Amsterdam Protocol of welfare and animal protection and National Institutes of Health, USA NIH, guidelines: Guide for the Care and Use of Laboratory Animals) and were approved by the local Institutional Animal Care and Use Committee at the School of Animal Science (University of La Plata). Anesthetized mice (intraperitoneal injection of 100 mg/kg ketamine and 5 mg/kg xylacine) were killed by cervical dislocation by experienced research personnel, which performed it humanely and effectively. All efforts were made to alleviate suffering during the whole experiment.

For the human sample analysis a written informed consent was obtained from parents of patients and the project was approved by the Ethics Committee of the Argentinean Association of Allergy and Clinical Immunology (February 2012). Sera were obtained from patients assisted at Hospital San Juan de Dios de La Plata.

### Statistical Analysis

All statistical analyses were carried out using GraphPad Prism 5 software. The significance of the difference was determined using an independent-sample t-test or ANOVA test. A p-value<0.05 was regarded as statistically significant.

## Results

### Beta conglycinin Alpha Subunit and the Polypeptide Fragment α-T are Specifically recognized by Different CMP specific Antibodies

**Figure 1:**
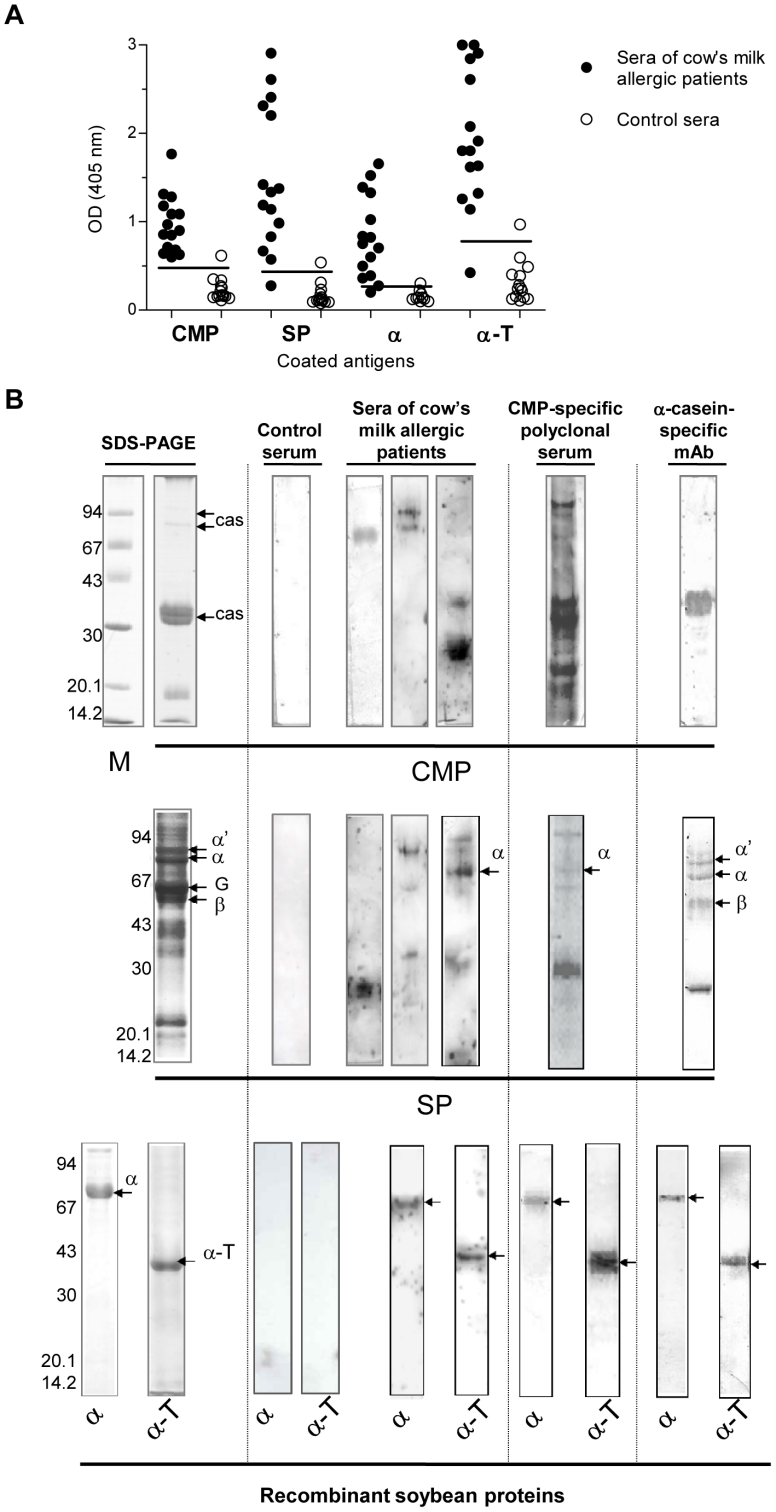
Immunochemical cross-reactivity. **A) Detection of specific human serum IgE antibodies by indirect ELISA.** Microtiter plates were coated with CMP, SP, recombinant α-subunit of β-conglycinin “α” or α-T, and sequentially incubated with sera of CMA patients and anti-human IgE specific conjugate. 15/15 sera contained IgE antibodies specific for CMP, 13/15 sera contained IgE antibodies specific for α and 14/15 sera contained IgE antibodies for α-T. **B) SDS-PAGE and immunoblotting of CMP and SP.** SDS-PAGE was performed under non reducing conditions for CMP (**upper panel**), SP (**middle panel**), and purified recombinant α and the α-T fraction (**lower panel**). Immunoblottings were developed with patient sera containing milk-specific IgE antibodies or control sera (from non-allergic patients) (1∶4), CMP-specific rabbit polyclonal antiserum (1∶1,000), and α-casein-specific monoclonal antibody (1D5 mAb 1∶1,500). *CMP: Cow's milk protein, cas: caseins, SP: soybean protein, G: 11S glycinin subunits; β-conglycinin subunits are indicated as α, α′ and β. M: Molecular masses are given on the left in kilo Daltons (kDa).*

We previously showed that although the soybean variety Raiden lacks the cross-reactive G4 glycinin, a residual cross-reactivity, probably due to the 7S seed globulin α-subunit of β-conglycinin (Gly m 5.0101), was detected [14]. In this work, recombinant Gly m 5.0101 (α) and a 235 amino acid residue polypeptide fragment (∼27 kDa) containing the three helix and one cupin domain, α-T, were immunochemically evaluated. Different CMP-specific antibodies were used to assess the cross-reactivity of these soy components (Figure 1). The amount of different coated antigens was adjusted in the indirect ELISA to have the same molar relationship. We found that all sera recognized CMP (100%), while most of the milk allergic patient sera presented IgE antibodies that recognized α (86.6%) and α-T (93.3%) (Figure 1A). The pattern reactivity obtained by immunoblotting revealed that human and rabbit polyclonal sera recognized caseins as monomers or aggregates (Figure 1B upper panel), whereas the 1D5 mAb bound α- casein. As it can be seen in the medium panel, these sera recognized soy components of 30 kDa and higher MW, corresponding to α, α′, and β subunit of β-conglycinin. The 30 kDa-band may correspond to the processed polypeptide A5A4B3 of G4 glycinin, previously described as cross-reactive protein [13], [14]. The lower panel shows that recombinant α and α-T were recognized by the different milk specific antibodies.

**Figure 2:**
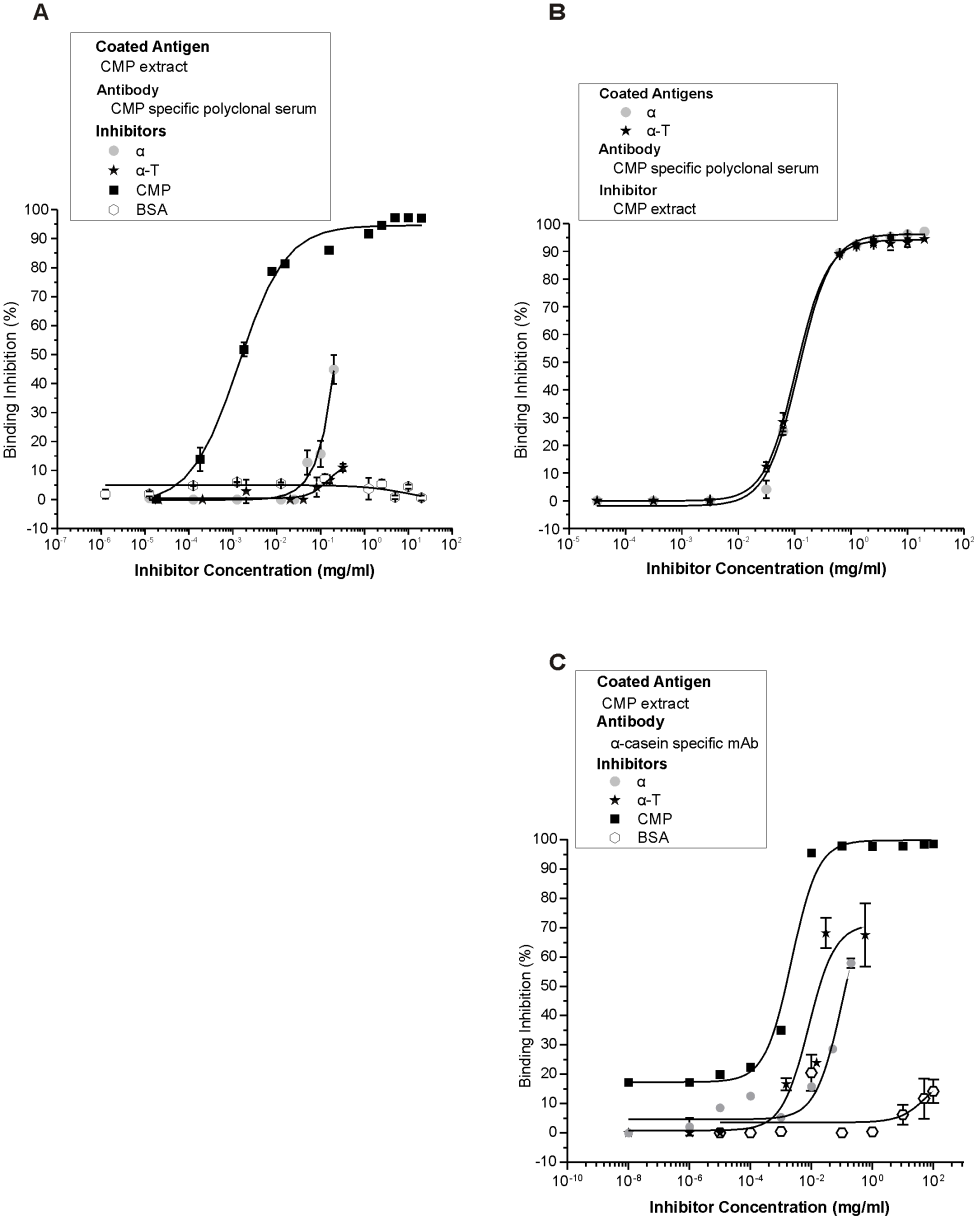
Sequential competitive ELISA with the CMP-specific rabbit polyclonal antiserum and 1D5 mAb. Microtiter plates were coated with CMP (1 µg/well) (**A** and **C**), α (0.1 µg/well) or α-T (0.1 µg/well) (**B**). CMP-specific polyclonal antiserum (**A**: 1∶200,000; **B**: 1∶6,000) or α-casein-specific mAb (**C**: 1∶300,000) were pre-incubated with different inhibitors at several concentrations and then added to Ag-coated wells. Then the appropriate conjugated secondary antibodies were added and the color was developed.

We next investigated the specificity of the antigen-antibody interaction with a sequential competitive ELISA. To enhance the sensitivity and the detection capacity of the assay, the CMP-specific antiserum or the mAb were pre-incubated with different concentrations of soluble inhibitors (reactant proteins). Then, soluble unbound CMP-specific antibodies were allowed to react with the coated antigen. The sigmoid shape of the dose-response curve obtained confirmed the specificity of the Ag-Ab binding (Figure 2). The binding of the CMP-specific polyclonal antiserum to the immobilized CMP was 100% inhibited with 3 mg/ml of soluble CMP, while 50% of inhibition (IC50) was achieved with 0.002 mg/ml CMP and 0.2 mg/ml α (Figure 2A). On the other hand, when any of the recombinant polypeptides was coated to the solid phase, sigmoid curves were obtained with a lower dilution of the polyclonal antiserum (1∶6,000), as compared with that used when CMP was coated to the solid phase. This assay rendered an IC50 of 0.1 mg/ml for CMP (Figure 2B). Taken together, and not surprisingly, CMP binding to milk-specific antibodies was more efficiently inhibited with CMP than with soybean components. However, the recombinant soy polypeptides could specifically inhibit the binding of the milk-specific antibodies to the coated homologous antigen. Figure 2C shows the sigmoid curves obtained with the α-casein-specific mAb used in the immunoblottings depicted in figure 1B. More than 95% of inhibition was achieved with a minimum of 0.01 mg/ml CMP as soluble inhibitor, while none of the other proteins could achieve such values at the range of the concentration assessed. The IC50 was 0.0015 mg/ml for CMP, 0.019 mg/ml for α-T and 0.13 mg/ml for α. A non-related control protein (BSA) rendered a constant basal inhibition of less than 10% in all assays (Figures 2A and C).

### Kinetic and Equilibrium Constants between α-casein-specific mAb 1D5 and Bovine α-casein and the Recombinant Soybean Proteins

The 1D5 mAb was employed to analyse the kinetic, equilibrium and affinity constants with α-casein and soy components (Table 1). According to the affinity constant (K_A_) value of 1.18×10^7^ M^−1^ for α-casein we consider that this antibody recognizes with a medium affinity its identity antigen. Interestingly, similar values were found for α and α-T. However, a lower K_A_ (4.06×10^6^ M^−1^) was measured for PA, which may be due to the smaller size of this fragment, and hence to fewer contacts with the antibody, as compared to α and α-T. Besides, a discrete increase in association constant (*k_ass_*) was observed for α as compared with its specific antigen, α-casein. This result may be explained by a different conformation or a different charge on the surface of the epitope in α, which produced more productive contacts with the 1D5 mAb.

**Table 1:** Kinetic and equilibrium constants for complexes formed by α-casein, α subunit of β-conglycinin (α), α-T and PA with the 1D5 mAb, specific for α-casein.

	k_ass_ (M^−1^ s^−1^)	k_diss_ (s^−1^)	K_A_ (M^−1^)	K_D_ (M^−1^)
Bovine α-casein	1.05×10^4^±0.14×10^4^	8.88×10^−4^±0.22×10^−4^	1.18×10^7^±0.13×10^7^	8.44×10^−8^±1.48×10^−8^
α	1.89×10^4^±0.18×10^4^	9.79×10^−4^±1.47×10^−4^	1.93×10^7^±0.22×10^7^	5.18×10^−8^±0.60×10^−8^
α-T	3.14×10^4^±0.44×10^4^	2.70×10^−3^±0.40×10^−3^	1.16×10^7^±0.06×10^7^	8.60×10^−8^±0.44×10^−8^
PA	6.92×10^3^±0,48×10^3^	1.70×10^−3^±1.15×10^−3^	4.06×10^6^±2.75×10^6^	2.46×10^−7^±1.67×10^−7^

*k_ass_*: Association constant, *k_diss_*: Dissociation constant, K_A_: Affinity constant, K_D_: Equilibrium Dissociation constant.

### Peptides containing Cross-reactive Epitopes were identified in α-T

**Figure 3:**
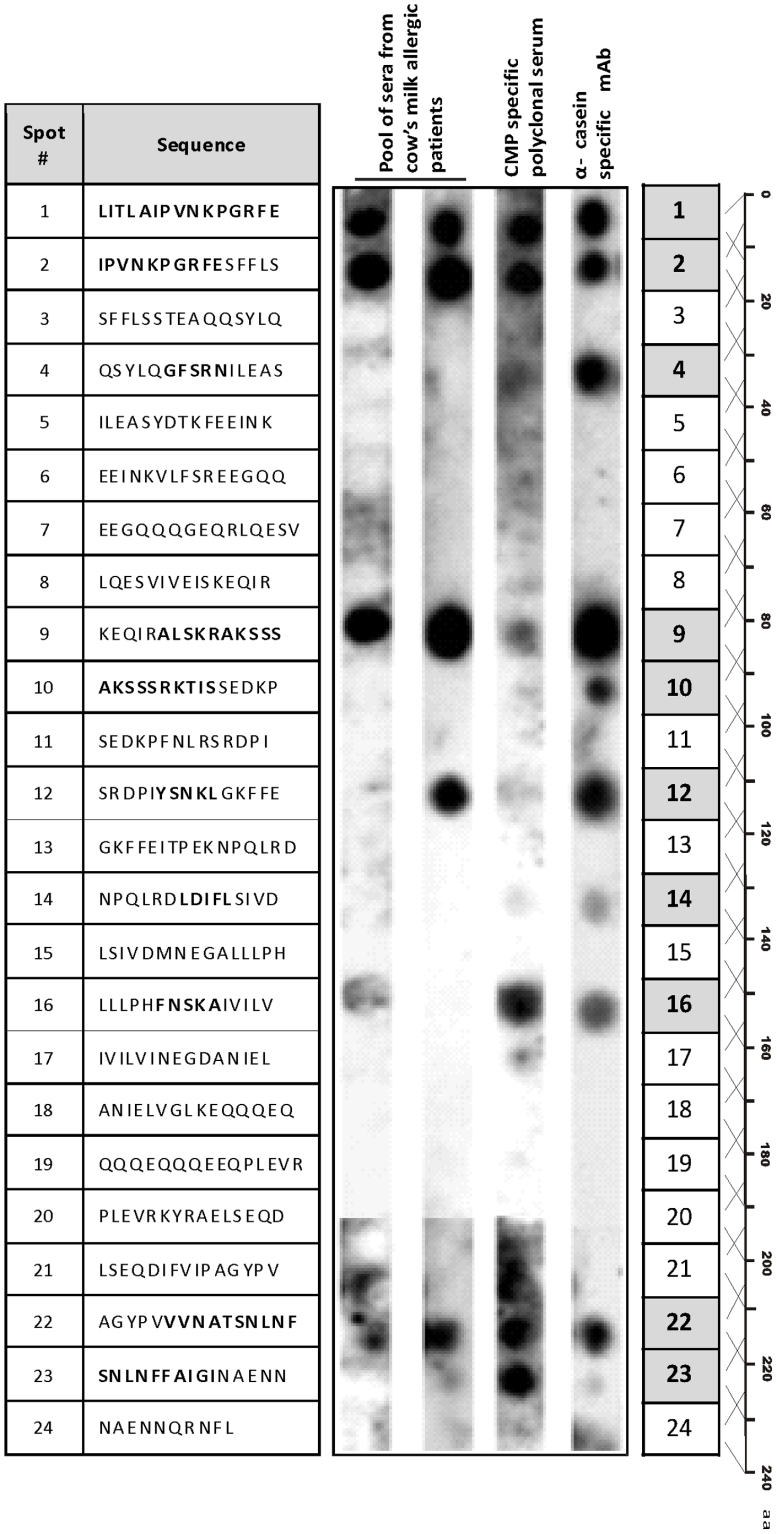
Epitope mapping of α-T. SPOTs array depicting the overlapping 15-aminoacid peptides covering the complete sequence of α-T, and probed with two different pools of serum from cow's milk allergic patients, a rabbit CMP-specific polyclonal serum and the 1D5 α-casein-specific mAb, followed with the appropriate conjugated secondary antibodies. Positive spots are shaded in grey boxes and recognized amino acids are bolded in the sequence depicted for spotted peptides.

Once we have detected that α-T contained B epitopes, 24 overlapping 15-mer peptides with 5 amino acid overlaps were synthesized on modified cellulose paper covering the whole sequence of α-T. Spots were probed with different CMP-specific antibodies to identify IgE and IgG cross-recognized zones (Figure 3). We observed similar reactivity patterns with the rabbit polyclonal antiserum and human sera: spots 1, 2 and 9 exhibited strong human IgE binding, while spots 1 and 2 showed a strong reactivity with the rabbit antiserum. We observed a faint reactivity on spots 16 and 22 with the pooled sera, and, on spots 9 and 12 with the rabbit antiserum. Peptides on spots 14 and 23 were only recognized by the rabbit antiserum. On the other hand, the α-casein-specific monoclonal antibody recognized peptides on spots 1, 2, 9, 12, 16 and 22 with high or middle intensity, and, unlike human IgE and rabbit IgG, it also recognized peptides on spots 4, 10 and 14. In summary, 10 peptides along the aminoacidic sequence of α-T were recognized by the different CMP-specific antibodies, indicating that cross-reactivity is not due to a single epitope (spots marked with grey boxes).

Furthermore, we calculated the net charge of the peptides and remarkably we found that reactive peptides are all positive, but peptide 14 (charge −2.2), whereas non reactive peptides are all negatively charged at pH 7.5 (Figure S1 A).

### *In Silico* Analysis showed that Conformational and Linear Epitopes are implicated in Cross-reactivity

**Figure 4:**
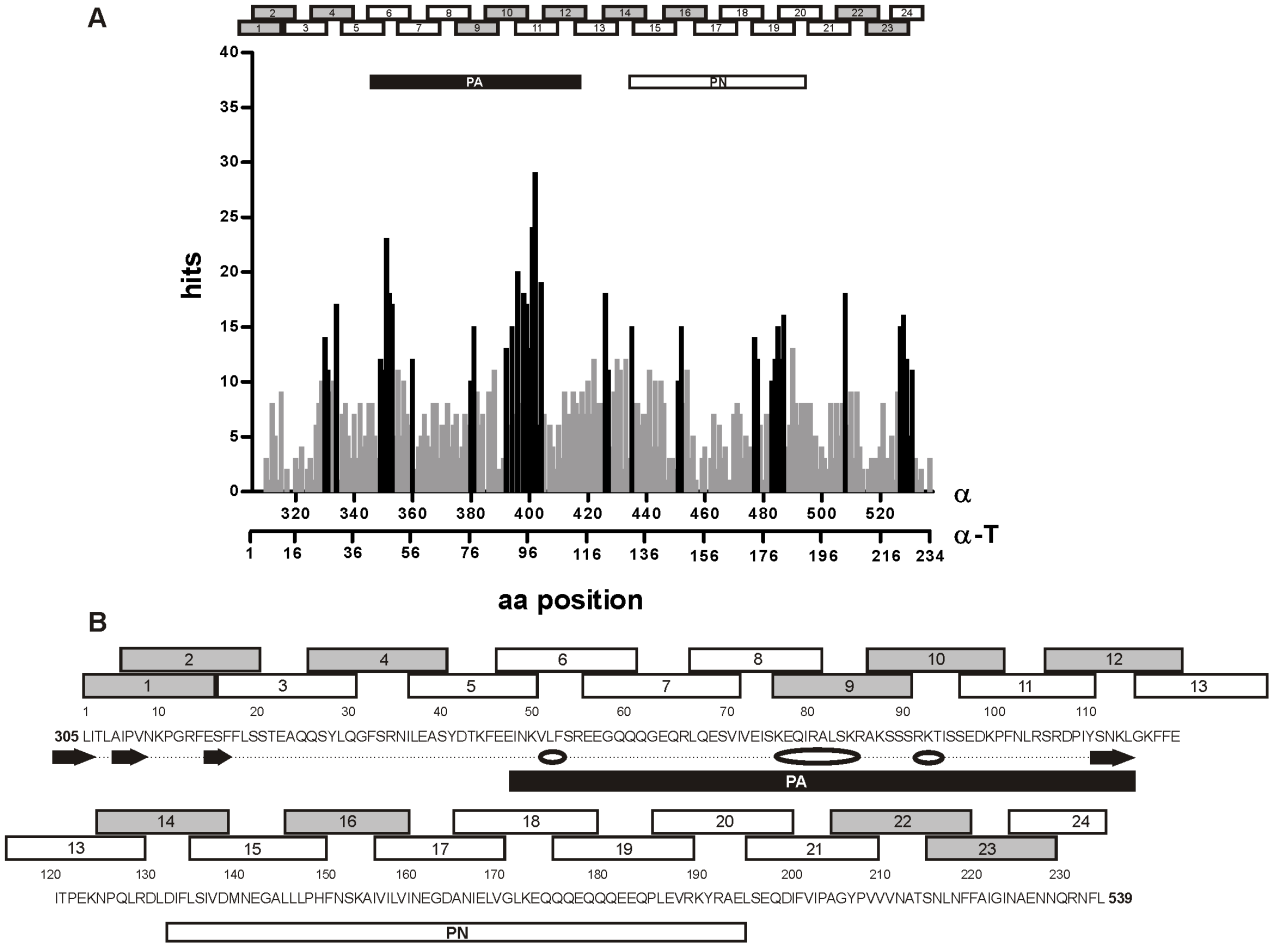
Schematic localization of the identified spots along the α-T sequence and sequence alignment analysis. **A**) Analysis of the sequence alignment of α-T with IgE epitopes in bovine caseins (IEDB) shown as hit distribution of amino acid similitude per position along the sequence of α-T. Positions of α-T with high hit values (black columns) correlate with positive spots of the overlapping assay (spots 9, 10 and12); **B**) The sequence of α-T (1–234 aa) is depicted along with overlapped peptides, PA and PN (305–539 aa correspond to α). *In silico* predicted alpha-helix (white ovals) and beta-sheet (black arrows) zones are indicated. PA contains the amino acid positions with the highest hits and the α-helix and β strand secondary structures.

In agreement with the epitope mapping of α-T done *in vitro* (Figure 3), sequence alignment analysis in figure 4A and figure S2 showed a high aminoacidic similitude (hits) in those positions where positive spots were obtained in the overlapping assay. High hit values along the α-T sequence where observed around amino acid positions 20 and 40 (region corresponding to positive spots 2 and 4), 77 and 90–100 (spots 9 and 10), 150 (spot 16), and 220 (spots 22 and 23). In figure 4B the sequence of α-T is depicted along with the peptides analyzed in the overlapping assay and the secondary structure predicted with different bioinformatics tools. As it can be observed, the selected PA peptide contains the region with the highest hits (peptides 9, 10 and 12).

The *in silico* predicted secondary structure (Figure 4B) showed that PA contains α-helix and β-sheet conformations. PN peptide was proposed as a control peptide since no secondary structure was predicted in this sequence and it contains low hit regions.

In addition, the amino acid composition was analyzed and we found hydrophilic (serine, asparagine, alanine and lysine) and hydrophobic amino acids (phenylalanine, isoleucine and leucine) as the most frequent amino acids in the different positive peptides in the overlapping assay of α-T. PA contains a compositional bias to hydrophilic amino acids such as glutamic acid (E), serine (S), lysine (K) and arginine (R) in the α-helix secondary structures (Figure S2).

**Figure 5:**
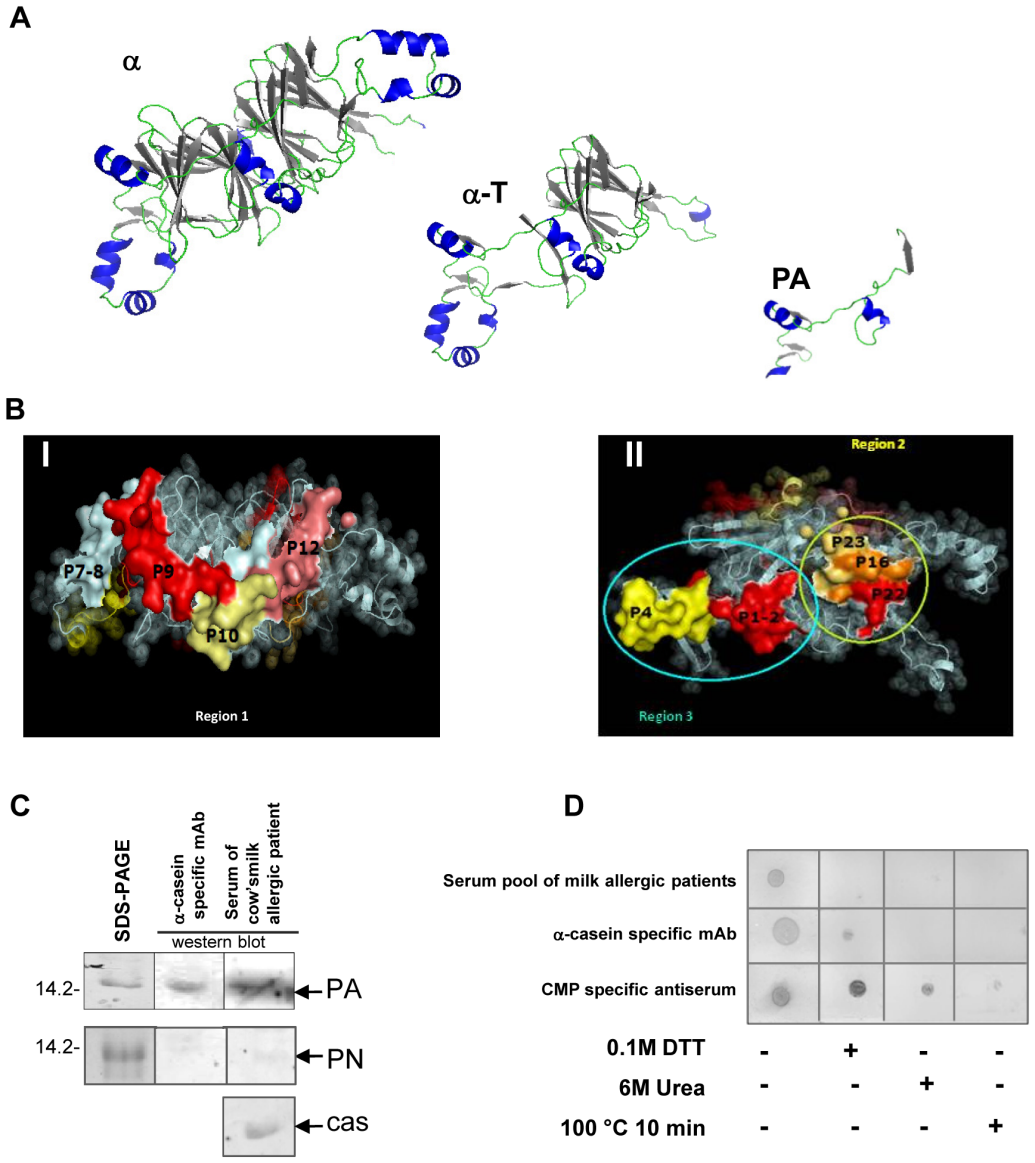
*In silico* and *in vitro* analysis of α, α-T and PA. **A**) Homology model obtained using the Modeller and visualized by PyMOL of α-subunit of β-conglycinin, α-T and PA, based on NCBI Conserved Domain Search results with α′ subunit as template (α-helices are shown in blue and β-sheets as dark grey arrows on the structure). **B**) Surface representation of positive overlapped peptides marked in different colors according to the intensity of the reaction (from pale yellow to red); I and II correspond to opposite sides of the α molecule. Positive peptides localized on the surface of the molecule seem to converge in three regions or putative epitopes. The remaining part of the molecule is represented in transparent spheres showing the secondary structure. **C**) SDS-PAGE of recombinant PA and PN and immunoblotting developed with 1D5 α-casein-specific mAb and a representative serum of CMA patients. Alpha-s1 casein was used as positive control (Molecular masses are shown on the left as kDa). **D**) Dot-blot of recombinant treated and untreated PA revealed with a pool of CMA patient sera, α-casein-specific mAb and CMP-specific polyclonal rabbit antiserum. Denaturing conditions consisted on: 0.1M DTT solution, 6M urea or 100°C for 10 minutes. *Cas: Alpha-s1 casein*.

Since conformational epitopes may be involved in this cross-recognition, homology models of α, α-T and PA were built (Figure 5A). As it can be seen in figure 5A the α-helix and β-sheet conformations are highly critical for the spatial distribution of all components. It became evident that peptides containing the cross-reactive domains are surface exposed and accessible to the solvent (Figure 5B).

The 3D models obtained were built using the structure of the soy α′ subunit of β-conglycinin from soybean (PDB 1UIK), which shows 90% identity with α subunit of β-conglycinin, as derived from fold assignment. The best structural model was evaluated using ProsaII and a Z-score = −7, indicating a high reliability.

According to the peptide reactivity and localization on the surface of the α subunit of β-conglycinin, three putative epitopes can be proposed (Figures 5B I and II). The region 1 would be contained in the PA fragment, while regions 2 and 3 could be structurally close, but on the opposite side of PA in the molecule.

Analyzing the secondary structure of the three immune reactive regions, and taking into account homology modeling results (Modeller) and the superficial residues visualized by PyMol, we found that α-helices, β-strands and connecting loops are relevant for the region 1-containing PA, which includes the positive spots 9, 10 and 12 of the overlapping assay. Instead, region 2 (contains P16, P22 and P23) and region 3 (contains P1, P2 and P4) would be mainly composed of superficial connecting loops. Furthermore, we studied the electrostatic potential on the surface of the three reactive regions using the PBEQ-Solver interface [35] on the web. The surface charge of the region 1 is positive (Figure S1B I) suggesting the predominance of electrostatic interactions with antibodies. On the other hand, the analysis of the electrostatic potential of the accessible solvent surface of region 2 and 3 (located on the opposite side of the molecule) (Figure S1B II) showed different features which may imply different interactions with antibodies. As judge for the electrostatic potential, surface epitopes contained in region 2 may be recognized by antibodies through hydrophobic or non-ionic interactions, while for the region 3 hydrophobic forces may be mainly involved, with a component of electrostatic bonds.

### Immunoassays showed the Presence of Linear and Conformational Epitopes on PA

Immunoblotting with different cow's milk-specific antibodies was assessed to validate the cross-reactivity of recombinant PA and PN. The immunoblotting pattern reactivity observed for PA indicates the presence of IgG- and IgE-binding epitopes, whereas PN showed no reactivity (Figure 5C). Elucidation of conformational epitopes involved in the cross-recognition of PA was assessed by dot-blot under different denaturing conditions (Figure 5D). Peptides heated or treated with urea and SDS showed a lower intensity when membranes were revealed with the CMP-specific antibodies (human sera containing IgE, the rabbit polyclonal antiserum and the mAb specific for α-casein), as compared to untreated PA. However, the reactivity observed with the rabbit polyclonal antiserum with DTT- and urea-treated PA led us to speculate that this antiserum also contains milk-specific antibodies capable of recognizing linear IgG epitopes.

### *In Vivo* and *In Vitro* Cross-reactivity of Soybean Components was confirmed with a CMA Murine Model

**Figure 6:**
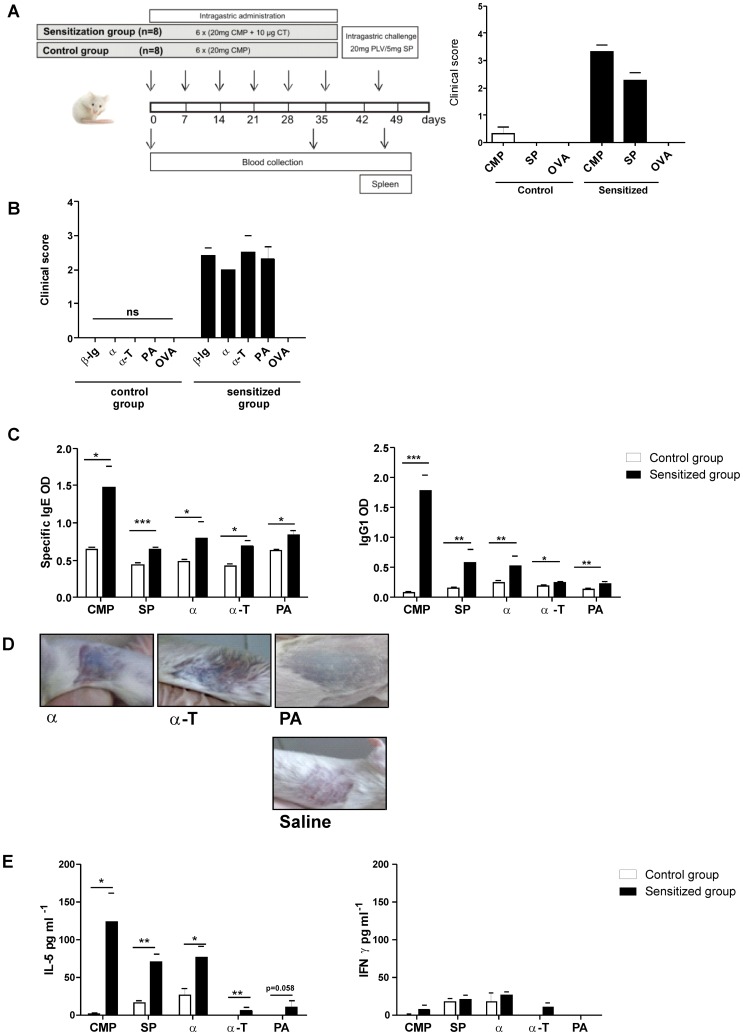
*In vivo* cross-reactivity assessed in a food allergy mouse model. **A**) Schematic representation of the experimental protocol: BALB/c mice were subjected to weekly intragastric sensitization with cholera toxin and CMP from day 0 through day 35. Challenge was performed at day 45 by ig administration of proteins (CMP, SP or OVA). Control mice only received CMP and then they were challenged with the different antigens. Symptoms were observed 30 min following the oral challenge and scored according to Table 2. **B**) Clinical scores assigned to symptoms observed 30 min following the sublingual challenge with 10 µg of β-lg, α, α-T, PA or OVA. **C**) Specific IgE and IgG1 in serum of milk-sensitized and control mice were assessed by EAST or indirect ELISA at day 45, respectively (mean values ± SEM). **D**) Cutaneous test performed in sensitized mice subcutaneously injected with 10 µg of antigens (α, α-T or PA) in the right flank and with saline in the left flank. The presence of blue color in the skin within minutes was considered a positive cutaneous test. **E**) Levels of IL-5 and IFN-γ assayed on stimulated splenocyte supernatants by ELISA (mean values ± SEM). Results correspond to a single experiment with five mice per condition, representative of three separate experiments that gave similar results. Statistically significant differences are denoted as starred values (*) ***p<0.005, **p<0.01, *p<0.05.

To further investigate the clinical relevance of the immunochemical cross-reactivity described, orally sensitized Balb/c mice with CMP were challenged with soy proteins (Figure 6A). Signs and symptoms were observed and scored on a scale from 0 to 5 according to the criteria shown in Table 2. We found that milk sensitized mice developed hypersensitivity symptoms immediately following the oral exposure to soy (SP, α, α-T and PA) or milk components (CMP and β-lg) (Figure 6A and B). No symptoms were observed in sensitized mice exposed to OVA, or in sham mice exposed to the different antigens. Remarkably, the sl challenge with individual proteins triggered immediate clinical signs (Figure 6B), thus reflecting the compromise of the mouth mucosa in animals that were intragastrically sensitized. These findings correlated with the high serum specific IgE levels (Figure 6C) and positive skin tests (Figure 6D) found in milk allergic mice injected with α, α-T and PA. Positive skin tests were also obtained with CMP and SP (pictures not shown). In addition, control mice rendered negative skin tests when injected with CMP, SP or soy recombinant components (pictures not showed). Every mice were injected with saline as control in the contra-lateral side and in all cases the reaction was negative (one is showed in Figure 6D). Moreover, Th2-associated IgG1 antibodies that were induced during the sensitization phase with cholera toxin, also recognized the different soy components. Specific IgG2a remained undetectable during the experiment (data not showed), indicating no Th1 induction during the sensitization phase.

**Table 2:** Clinical scores assigned to symptoms triggered following the oral or sublingual challenge.

Score	Symptoms
0	No symptoms
1	Scratching and rubbing around the snout and head
2	Puffiness around the eyes and mouth, pilar-erecti, diarrhea, reduced activity and/or decreased activity with increased respiratory rate
3	Respiratory distress, cyanosis around snout and tail
4	No activity upon stimuli, convulsion
5	Death

Finally, we found an increased secretion of IL-5 by spleen cells stimulated with CMP, SP, α and α-T (Figure 6E). PA showed a slight increase in the production of this Th2-associated cytokine, while IFN-γ remained low, as expected. These findings clearly indicate that CMP-specific T cells elicited during the sensitization phase can be *in vitro* stimulated with soy components.

## Discussion

The characterization of the cross-reactivity between milk and soy allergens may shed light on the structural and immunologic relationships between phylogenetically unrelated allergens. Our recent studies show that members of the 11S (Gly m 6) and 7S (Gly m 5) globulin storage soy proteins are cross-reactive allergens with bovine caseins [13]–[15]. The immunochemical cross-reactivity described has been biologically confirmed using an IgE-mediated mouse model of food allergy with mice exclusively sensitized to CMP [15]. This finding highlights the potential clinical relevance of this phenomenon in allergic patients, although limitations are obvious with conclusions extrapolated from animal models. However, we consider they provide a useful biological tool when ethics is concerning, and in situations in which co-sensitization in patients is almost impossible to disprove. There are some studies reporting the occurrence of clinical intolerance to soy-based formula in milk allergic patients without previous sensitization to soy allergens [9], [10]. Although they show that it occurs in a small proportion of patients (<15%), this intolerance is a clinical common concern in our population. Milk allergic patients may develop hypersensitivity reactions following accidental exposure to soy after the ingestion of soy-containing foods, or during the restrictive treatment with soy-based formula.

In this study, we have focused on investigating shared B and T epitopes of Gly m 5 with bovine caseins, which are relevant allergens of soy and milk respectively [11], [20]. Of note, the soy recombinant components were recognized as soluble antigens by a rabbit CMP-specific polyclonal antiserum and the α-casein specific monoclonal antibody in a competitive ELISA. These findings discard the possibility of neo epitopes created during the coating of antigens to the solid phase as responsible for this cross-reactivity and confirm that α and α-T contain cross-reactive B epitopes. This assay rendered a higher IC50 for α than that for CMP with the polyclonal and monoclonal antibodies (150 and 86 times respectively), which is reasonable since these antibodies are primary specific for milk components. These results and the shape of the dose-response curves indicate that the antigen-antibody interaction is specific, that the α protein and its fraction α-T bear cross-reactive epitopes with bovine α-casein, and that a restricted population of CMP-specific antibodies specifically recognized B epitopes in α and α-T polypeptides. These findings were further characterized with the biosensor binding assay, which showed that α-casein, α and α-T present similar affinity constant to the α-casein-specific mAb. The higher *k_ass_* value found for α-T as compared with that for α and α-casein can be due to stronger favourable contacts in the interface formed between mAb 1D5 and α-T. However, the *k_diss_* value means that this complex is more unstable than those formed with α and α-casein. Likewise, the smaller *k_ass_* value found for PA as compared to the other components analyzed can be explained by lower contacts with 1D5, which may be due to its smaller size and conformational changes. As expected, we found medium affinities for this monoclonal antibody with different cross-reactive antigens, which may reflect an overall approach to understand the cross-reactivity implicated in a hypersensitivity reaction. Recently, and using synthetic allergens, it has been shown that IgE antibodies do not necessarily need to have a high affinity with its specific antigen to trigger mast cell degranulation [41]. In this study we have shown that even a 64 amino acid residue peptide of moderate affinity triggers a specific allergic response in milk allergic mice, thus indicating that it can activate sensitized cells.

To gain more insight into the molecular feature of this cross-reactivity we next characterized B epitopes (IgG and IgE) on different peptides of α (α-T and PA) using overlapping synthetic peptides, recombinant allergenic fragments and unfolded fragments. Besides, the overlapped peptide scanning revealed that cross-reactivity is not due to a unique shared epitope. The overlapping peptide assay showed that the epitope-bearing 15-mer peptides contain 33.8% of hydrophobic (A: alanine, F: phenylalanine and L: leucine), 29% of neutral hydrophilic (S: serine and N: asparagine) and 9.6% of charged hydrophilic aminoacids (K: lysine, E: glutamic acid) with a positive net charge, suggesting that different ways of antibody-antigen recognition may be possible in this type of cross-reactivity (Figure S1). Since the combining site of an antibody may present different zones with different charges, and furthermore, different conformers of the paratope can be found in unbound antibodies exhibiting different surfaces of interaction with antigens in this regard, a single antibody can interact with different epitopes even on the same antigen. In the particular case of the PA, which is mainly positively charged, the interactions with antibodies are expected to be electrostatic with a net negatively charged paratope. Regarding epitopes present in the region 2, the type of interactions are mainly hydrophobic, but analyzing epitopes in the region 3 those interaction could be a combination of hydrophobic and electrostatic ones. Thus, the studied cross-reactivity implies a more complex interpretation than just considering the presence of a unique and shared epitope.

The *in silico* analysis for prediction of cross-reactive epitopes, secondary structure and modeling of Gly m 5 indicated that PA contains cross-recognized conformational and linear epitopes that are exposed on the surface of the allergen to interact with antibodies. Then, IgG and IgE cross-reactive epitopes were confirmed in PA using different milk-specific antibodies and immunoassays. It has been described that the feature of the epitope in caseins is critical for the history of milk allergy in allergic patients [42]–[44], and the recognition of conformational epitopes is a predictive factor for a later acquisition of tolerance to CMP. Therefore, monitoring the reactivity of serum antibodies against PA might constitute an additional prognostic tool for achievement of immune tolerance in patients. Although the disruption of dominant epitopes in PA using chaotropic agents provided preliminary data, further investigations with structural biology techniques such as X-ray crystallography and nuclear magnetic resonance are necessary to visualize these conformational cross-reactive epitopes. In this sense, the combination of different mapping techniques with the visualization of the 3D models is needed to assess the structural integrity of allergens. Food antigens, which have mainly linear epitopes following food processing or digestion, also contain conformational epitopes that can be affected by food processing, thus interfering with the conformation, IgE binding activity and hence the allergenicity.

The use of different sequence databases of caseins allergenic epitopes provided no information of shared sequences with the soy component studied here. However, the sequence database obtained by us, using the describe techniques above, provided of several peptides of α-subunit of β-conglycinin that presented homology with milk caseins and their cross-reactivity was *in vitro* confirmed. Therefore, based on *in silico* and experimental data we have selected a peptide from the sequence of α-T (PA) that matched the identified IgE-binding epitopes in caseins [42], [45], [46]. Fu et al [47] identified IgG sequential and solvent-exposed epitopes (185–231) in the N-terminus domain of the α-subunit of the β-conglycinin, which is not coincident with the epitope here described in the C-terminus domain. Therefore this peptide of Gly m 5 represents a new IgE-binding epitope that occurs in a conformational region of the protein exposed to the solvent.

In this study, the combination of bioinformatics tools for prediction of epitopes, secondary structure, surface electrostatic charge, sequence alignment, molecular modeling and visualization of immunodominant regions in 3D modeled molecules, with immunochemical techniques (immunoblotting, indirect ELISA and competitive ELISA), SPR biosensor and *in vivo* experiments, allowed us to strongly demonstrate the existence of cross-reactivity between Gly m 5 and caseins. Besides, we have selected a candidate peptide that contains linear and conformational B, and T epitopes, which could be easily rendered hypoallergenic for a potential and safe therapeutic treatment.

The clinical symptoms elicited after the sublingual challenge with α, α-T and PA, and the positive skin prick tests confirmed the presence of at least two IgE-binding epitopes on the Gly m 5.0101 soy fractions. Of note, the presence of linear T epitopes could be evidenced when spleen cells were stimulated with these fractions, with the secretion of IL-5. The ability of α, α-T and mainly PA for T cell activation encourage us to propose them in a potential mucosal therapy for tolerance induction in patients with milk and/or soy allergy.

As a conclusion, using *in vitro*, *in vivo* and *in silico* approaches we have identified a peptide of Gly m 5 that contains B and T cross-reactive epitopes with bovine casein. This information sheds light on the characterization of this relevant soybean allergen, and may provide a molecular explanation for the complex IgE cross-reactivity between the phylogenetically unrelated milk and soy allergens. This phenomenon may explain the secondary soy allergy elicited during the treatment of some cow's milk allergic patients. Finally, this knowledge may be exploited to develop a mucosal tolerizing immunotherapy using PA for intervention in soy and milk allergy.

## Supporting Information

Figure S1 Electrostatic and charge analysis of α-T. **A**) pI and charge at pH = 7.5 for peptides of the overlapping assay estimated with the Protein Calculator v3.3 (http://www.scripps.edu/~cdputnam/protcalc.html). **B**) Electrostatic potential surface on the α subunit of β-conglycinin showing **I**) region 1, which contains the PA fragment, and **II**) regions 2 and 3. Note that the region 1 is mostly positively charged, region 2 is mostly hydrophobic and region 3 is hydrophobic and slightly positively charged.
(TIF)

Figure S2 Multiple sequence alignment between sequences of α-T and epitopes on bovine casein peptides. A total of 73 peptides of α-S1 (1–55), α-S2 (56–63), β (64–67) and κ (68–73) bovine caseins, described as T and/or B epitopes (IEDB, (http://www.immuneepitope.org/) were aligned with α-T sequence using the ClustalW2 server (http://www.ebi.ac.uk/Tools/msa/clustalw2/). Common amino acids are shaded in red on casein peptide sequences, or in grey in the α-T sequence. Blue rectangles delimit the highest frequencies of similitude observed (which correspond to the high hit values in Figure 4 A). Green and yellow boxes underneath the α-T sequence correspond to PA and PN respectively.
(TIF)
