# Supplementary figures and images for: Targeting a Cross-Reactive Gly m 5 Soy Peptide as Responsible for Hypersensitivity Reactions in a Milk Allergy Mouse Model
Source: PLoS One. 2014 Jan 9;9(1):e82341. doi: 10.1371/journal.pone.0082341 (PMC3886974; doi:10.1371/journal.pone.0082341)

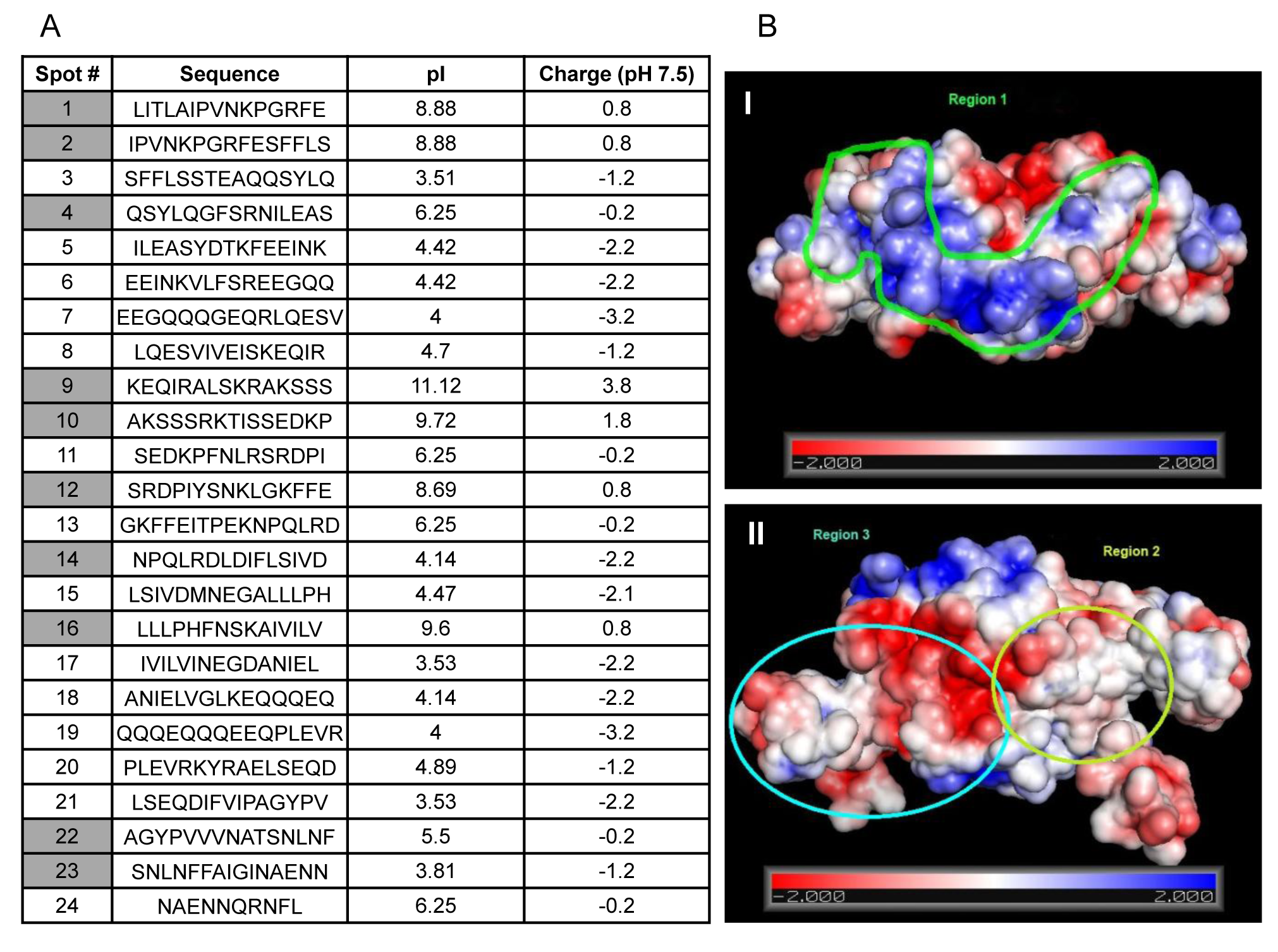

Supplement: Figure S1 — Electrostatic and charge analysis of α-T. A) pI and charge at pH = 7.5 for peptides of the overlapping assay estimated with the Protein Calculator v3.3 (http://www.scripps.edu/~cdputnam/protcalc.html). B) Electrostatic potential surface on the α subunit of β-conglycinin showing I) region 1, which contains the PA fragment, and II) regions 2 and 3. Note that the region 1 is mostly positively charged, region 2 is mostly hydrophobic and region 3 is hydrophobic and slightly positively charged. (TIF) [file pone.0082341.s001.tif]

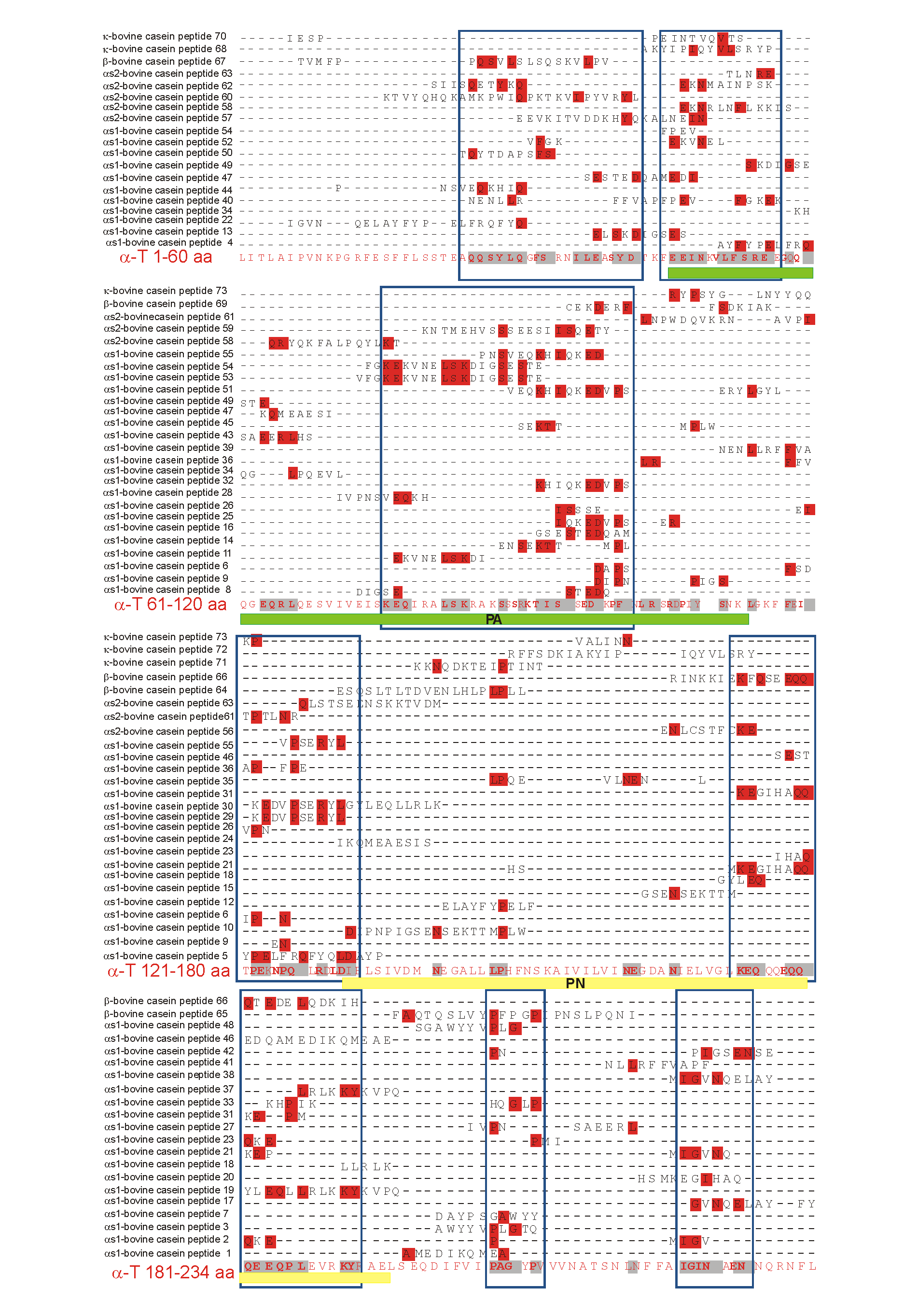

Supplement: Figure S2 — Multiple sequence alignment between sequences of α-T and epitopes on bovine casein peptides. A total of 73 peptides of α-S1 (1–55), α-S2 (56–63), β (64–67) and κ (68–73) bovine caseins, described as T and/or B epitopes (IEDB, (http://www.immuneepitope.org/) were aligned with α-T sequence using the ClustalW2 server (http://www.ebi.ac.uk/Tools/msa/clustalw2/). Common amino acids are shaded in red on casein peptide sequences, or in grey in the α-T sequence. Blue rectangles delimit the highest frequencies of similitude observed (which correspond to the high hit values in Figure 4 A). Green and yellow boxes underneath the α-T sequence correspond to PA and PN respectively. (TIF) [file pone.0082341.s002.tif]
